# Neuroimaging evaluation of high dose methotrexate-induced neurotoxicity in pediatric and young adults: a PET/MRI study

**DOI:** 10.3389/fnimg.2025.1659480

**Published:** 2026-01-13

**Authors:** Zahra Shokri Varniab, Hyun Gi Kim, Ricarda von Krüchten, Yashas Ullas Lokesha, Kristina Elizabeth Hawk, Shashi Bhushan Singh, Tie Liang, Sarah Lu-Liang, Lucia Baratto, Michael Iv, Heike Elisabeth Daldrup-Link

**Affiliations:** 1 Molecular Imaging Program at Stanford (MIPS), Department of Radiology, Stanford University, Stanford, CA, United States; 2 Department of Radiology, Eunpyeong St. Mary’s Hospital, College of Medicine, The Catholic University of Korea, Seoul, Republic of Korea; 3 Division of Neuroimaging and Neurointervention, Department of Radiology, Stanford University, Stanford, CA, United States; 4 Department of Pediatrics, Pediatric Oncology, Stanford University, Stanford, CA, United States

**Keywords:** cancer survivors, methotrexate, neurotoxicity, pediatric oncology, PET

## Abstract

**Introduction:**

High-dose Methotrexate (HDMTX) can induce neurotoxicity, yet its impact on brain metabolism remains underexplored. This study aimed to assess short- and long-term brain metabolic changes post-HDMTX on 18F-FDG PET/MRI relative to baseline (pre-HDMTX) scans.

**Methods:**

In this IRB approved, retrospective study, we included 19 children and young adults (3 females and 16 males; age 17.9 ± 4.3 years), with lymphoma (*n* = 13) or osteosarcoma (*n* = 6). All patients underwent 18F-FDG PET/MRI before (baseline) and after HDMTX (>1000 mg/m^2^). Post-treatment scans were conducted ≤3 months (short-term group, *n* = 11) or >3 months (long-term group, *n* = 8) after completion of HDMTX and were compared with baseline scans. SUVmean and SUVmax of the whole brain cortex and six subregions were measured with PMOD software. A generalized linear regression model was used to evaluate post-pre-HDMTX SUV values differences in whole cortex with *p* < 0.05 and for with of different brain subregions, with *p* < 0.008 after Bonferroni correction.

**Results:**

In the short-term group, compared with baseline, both SUVmean (pre-HDMTX vs. post-HDMTX: 5.06 ± 1.62 vs. 6.31 ± 1.71, *p* < 0.001) and SUVmax (9.16 ± 3.33 vs. 13.25 ± 3.35, *p* < 0.001) significantly increased in the whole cortex following HDMTX. In contrast, the long-term group showed no significant changes in SUVmean (6.31 ± 1.71 vs. 6.30 ± 1.54, *p* = 0.1) or SUVmax (12.01 ± 3.53 vs. 11.58 ± 3.07, *p* = 0.1) after HDMTX.

**Discussion:**

^18^F-FDG PET/MRI revealed short-term increases in brain metabolism post-HDMTX compared with baseline, possibly reflecting neuroinflammation. Long-term follow up scans revealed normalization of brain metabolism or decreased brain metabolism compared to baseline, the latter possibly indicating neurotoxicity.

## 1 Introduction

High-dose methotrexate (HDMTX) chemotherapy, defined as a cumulative methotrexate dose of more than 1,000 mg/m^2^ (Ackland and Schilsky, 1987; Jones et al., 2024), is used to treat patients with acute lymphoblastic leukemia, non-Hodgkin lymphoma, and osteosarcoma (Baratto et al., 2024). HDMTX is associated with significant neurotoxicity, including stroke-like symptoms, encephalopathy, seizures, headache, lethargy, altered mental status, blurred vision, and/or aphasia (Bhojwani et al., 2014; Inaba et al., 2008). Transient acute neurotoxicity can occur within days after MTX administration and often resolve (Inaba et al., 2008; Rubnitz et al., 1998). Previous studies (Peled et al., 2022; Inaba et al., 2008) showed acute neurological side effects, such as generalized seizures, confusion, encephalopathy, dysarthria, and choreiform movements, in pediatric patients with cancer at 2–10 days after HDMTX therapy.

By contrast, late effects of MTX develop slowly over time and persist, leading to long-term cognitive problems (Santangelo et al., 2022; Bhojwani et al., 2014).

Berlin et al. (2020) reported that HDMTX induced long-term cognitive impairment in a rodent model 16 months after treatment. Several authors described that long term neurotoxic effects after HDMTX can be attributed to a cascading mechanism that begins with microglial activation (Gibson et al., 2019; Seigers et al., 2010; Vazi et al., 2021). Taha et al. (2023) reported that activated microglia release proinflammatory cytokines and reactive oxygen species, leading to subsequent neuroinflammation and contributing to neuronal dysfunction and damage. Seigers et al. (2010) reported that MTX administration in rats resulted in reduced hippocampal blood vessel density and microglia activation. Gibson et al. (2019) reported that sustained microglial activation after HDMTX therapy contributed to neuronal damage and exacerbated long-term neurocognitive deficits in cancer survivors.

While our understanding of the pathogenesis of MTX-induced neurotoxicity has improved, studies investigating microglia activation and neuronal damage have primarily relied on immunohistochemical markers (Gibson et al., 2019; Seigers et al., 2010; Vazi et al., 2021). Investigators used staining for glial fibrillary acidic protein (GFAP) (Vazi et al., 2021) to assess astrocyte activation or Iba1 (ionized calcium binding adaptor molecule 1) (Seigers et al., 2010) for microglia in various rat brain regions.

Medical imaging studies investigated the effect of HDMTX on the brain at single time points after HDMTX therapy. Baratto et al. (2024) reported decreased brain metabolism in the cingulum and prefrontal cortex on 18F-FDG PET scans at 3.5 ± 1.5 months after HDMTX therapy, which correlated with reduced executive function scores in pediatric cancer survivors. Ponto et al. (2015) reported decreased brain metabolism of the bilateral orbital frontal gyri, right substantia nigra, and brainstem on 18F-FDG PET scans of breast cancer survivors which correlated with cognitive impairments in executive function, working memory, and divided attention. In addition, Baratto et al. (2024) reported significantly reduced cerebral blood flow (CBF) in the prefrontal cortex, cingulate gyrus, and hippocampus in pediatric cancer survivors after HDMTX treatment on MRI. Bansal et al. (2025) reported reductions in CBF of the prefrontal, temporal, parietal lobes and thalamus in pediatric acute lymphoblastic leukemia patients on MRI scans after HDMTX. However, longitudinal assessments of brain metabolism at various intervals following the completion of HDMTX therapy remain unexplored.

We hypothesized that HDMTX will result in short-term increases in 18F-FDG cortical metabolism, reflecting the inflammatory response described in preclinical investigations (Seigers et al., 2010), followed by decline in brain metabolism on long-term follow-up studies, indicating either resolution of microglia activation or progression to neuronal damage. Therefore, the purpose of our study was to assess long-term brain metabolic changes post-HDMTX on 18F-FDG PET/MRI scans.

## 2 Methods

### 2.1 Patients

**Figure 1:**
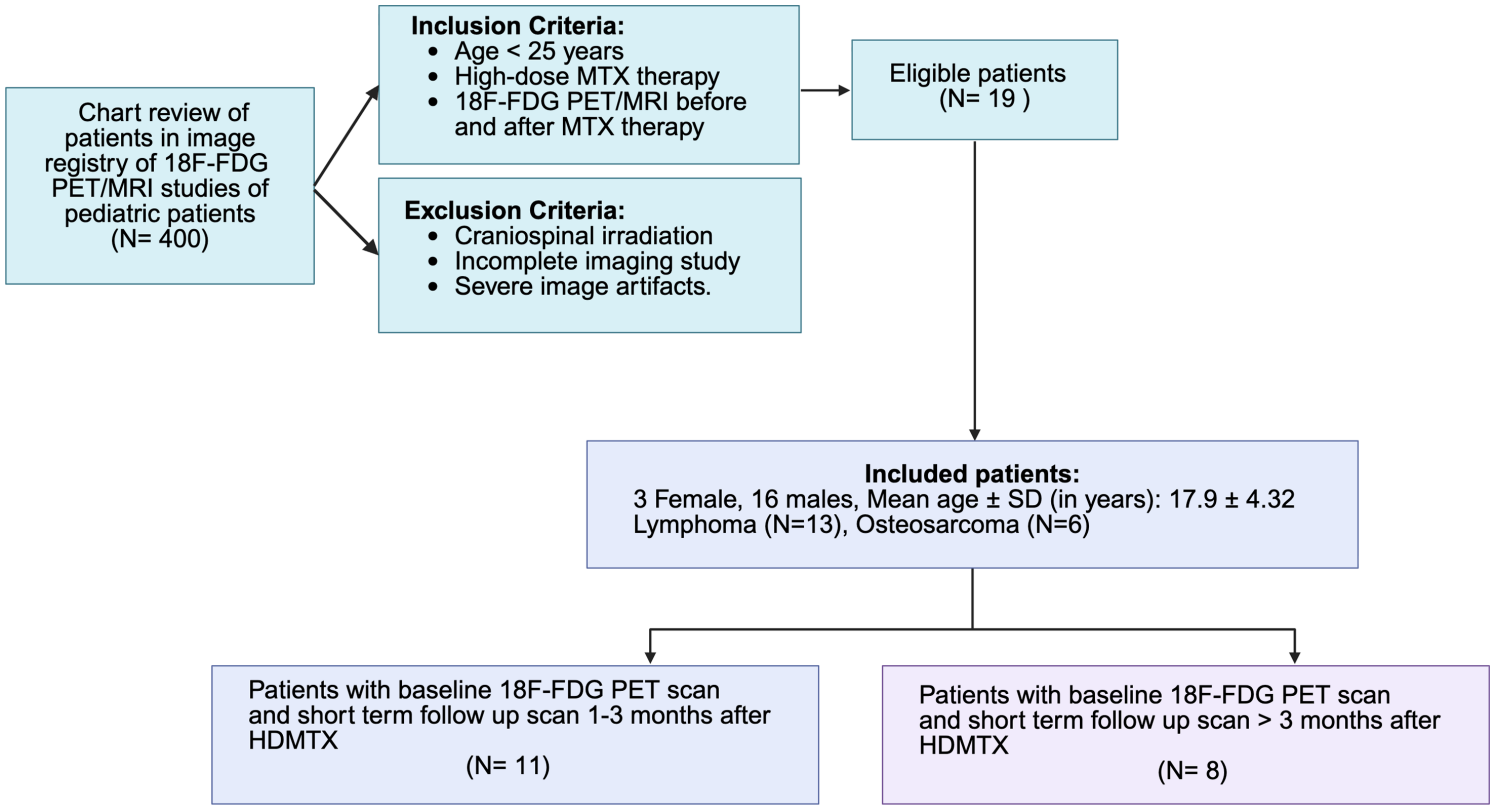
Patient selection flowchart. Patients were selected based on a centralized image registry of 400 ^18^F-FDG PET/MRI scans of pediatric cancer patients. The inclusion criteria required that patients be under 25 years of age, had undergone high-dose methotrexate (HD-MTX) therapy, and had both pre- and post-treatment ^18^F-FDG PET/MRI scans. Exclusion criteria included prior craniospinal irradiation, incomplete imaging studies, or severe image artifacts. Nineteen eligible patients (3 females, 16 males; mean age 17.9 ± 4.32 years) were included in the study, with diagnoses of lymphoma (*n* = 13) or osteosarcoma (*n* = 6). The cohort was further stratified into two groups: patients who underwent ^18^F-FDG PET/MRI scans at 1–3 months after completion of HDMTX (short-term follow-up, *n* = 11) and patients who underwent ^18^F-FDG PET/MRI scans at > 3 months after completion of HDMTX (long-term follow-up, *n* = 9).

This retrospective study was approved by the Institutional Review Board (IRB). The study included patients with history of HDMTX and 18F-FDG PET/MRI between April 2016 and August 2024 in a single institution. Inclusion criteria included: (1) Histopathologically confirmed diagnosis of lymphoma, leukemia, or osteosarcoma; (2) HDMTX therapy with a cumulative dose of 1,000 mg/m^2^ or more; (3) baseline and post-treatment 18F-FDG PET brain scans; and (4) age under 25 years. Exclusion criteria were (1) any prior cranial or craniospinal irradiation (including focal brain radiotherapy) to avoid confounding treatment-related effects on brain metabolism, and (2) incomplete imaging studies or severe image artifacts. Of 216 patients who underwent HD-MTX, 19 pediatric and young adult patients met the inclusion criteria (Figure 1). These included three females and 16 males with a mean age 17.9 ± (SD) 4.32 years (range 8–23 years) and a diagnosis of lymphoma (*n* = 13) or osteosarcoma (*n* = 6). Fourteen patients were Non-Hispanic/Non-Latino, and 5 were Hispanic/Latino. In terms of race, 7 patients were White, 5 were Asian, 4 were classified as Other, and 2 were Black or African American (Table 1).

**Table 1:** Patient demographics, cancer diagnosis, and treatment characteristics.

Characteristics	All patients (total *n* = 19)	Short-term follow-up ≤ 3 months (*n* = 11)	Long-term follow-up >3 months (*n* = 8)
Age at baseline PET scan, years, median (IQR)	19 (14–20)	18 (12–19)	20 (17–22)
Sex, *n* (%)			
Male	16 (83.2%)	9 (81.8%)	7 (87.5%)
Female	3 (15.8%)	2 (18.2%)	1 (12.5%)
Ethnicity, *n* (%)			
Hispanic/Latino	5 (26.3%)	3 (27.2%)	2 (25%)
Non-Hispanic/Non-Latino	14 (73.6%)	8 (72.7%)	6 (75%)
Race, *n* (%)			
White	7 (37%)	5 (45%)	2 (25%)
Asian	5 (26%)	2 (18%)	3 (38%)
Black or African American	2 (11%)	1 (9%)	1 (12%)
Other^a^	5 (26%)	3 (27%)	2 (25%)
Underlying malignancy, *n* (%)			
Lymphoma	13 (68.4%)	11 (100%)	2 (25%)
Osteosarcoma	6 (31.6%)	0 (0%)	6 (75%)
Cumulative HDMTX dose (mg): median (IQR)	36,000 mg (22,650–121,000 mg)	31,000 mg (21,500–37,500)	174,000 mg (35,000–240,000)

HDMTX, high-dose methotrexate.

a Other race includes American Indian or Alaska Native, Native Hawaiian or other Pacific Islander, individuals reporting more than one race, and those whose race was recorded as “other” or unknown.

**Figure 2:**
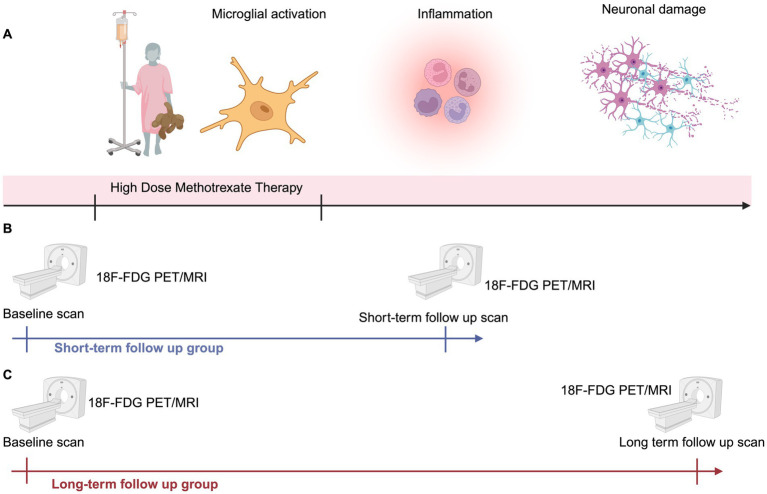
Study design. **(A)** HDMTX therapy induces microglial activation and potential neuronal damage. **(B)** Group 1 (short-term follow up group; *n* = 11) underwent ^18^F-FDG PET at baseline and at 1–3 months after completion of HDMTX. **(C)** Group 2 (long-term follow up group; *n* = 8) underwent ^18^F-FDG PET at baseline and at > 3 after completion of HDMTX.

We divided our patients into two groups: Short-term group: Eleven patients [2 females, 9 males; mean age 16 ± (SD) 4.3 years] underwent a pre-treatment 18F-FDG PET scan and a follow up scan within 3 months [1.5 ± (SD) 0.7 months] of completing HDMTX therapy. Long-term group: Eight patients [1 female, 7 males; mean age 19 ± (SD) 4.1 years] underwent a pre-treatment 18F-FDG PET scan and a follow up scan more than 3 months after completing therapy [11 ± (SD) 4 months]. The 3-month cutoff was pre-specified based on prior imaging and clinical reports of HDMTX-related brain injury, which describe an acute/subacute phase within days to weeks and delayed white-matter changes evolving over approximately 3–4 months after treatment (Baratto et al., 2024). We therefore defined scans obtained ≤3 months after the end of HDMTX as “short-term” and scans obtained >3 months as “long-term”. All patients in both groups received leucovorin (15 mg/m^2^/dose every 6 h) starting 24 h after the initiation of HDMTX, continuing until methotrexate levels were reduced to <0.1 μM (Figure 2). One of the patients, a 20-year-old male with diffuse large B-cell lymphoma (DLBCL), underwent both short-term (31 days) and long-term (18 months) 18F-FDG PET scans after HDMTX therapy. We included this patient in the short-term group using only the first scan. We included this patient as an independent case study and documented the longitudinal findings. For each PET scan time point, we retrospectively reviewed neurology and oncology notes to identify clinical evidence of neurotoxicity. Specifically, we recorded the occurrence of seizures, stroke-like focal neurological deficits including hemiparesis, facial droop, aphasia, encephalopathy or altered mental status, ataxia, movement disorders, and visual disturbances, as well as milder symptoms such as headache, dizziness, nausea, fatigue, and memory complaints.

### 2.2 Imaging protocol

All scans were performed using a 3T PET/MRI system (Signa; General Electric Healthcare). Patients fasted for at least 4 h before the 18F-FDG PET scan, and serum glucose levels were confirmed to be below 120 mg/dL at the time of 18F-FDG injection. A standardized dose of 18F-FDG (3 MBq/kg) was administered intravenously, adhering to the guidelines of the Society of Nuclear Medicine and Molecular Imaging and the European Association of Nuclear Medicine (Vali et al., 2021). During the uptake time, subjects were kept in a dimly lit room, instructed to rest with their eyes closed to reduce external stimuli. No sedation was administered. Acquisition of PET images began 60 min post-injection. 18F-FDG PET images were acquired in the axial plane with a field of view (FOV) of 60 cm, a matrix size of 192 × 192, and a slice thickness of 2.78 mm. For attenuation correction of 18F-FDG-PET data, non-breathhold axial Dixon sequences were acquired (TR/TE: 3.8/1.6 ms, FOV: 60 cm, slice thickness: 2.78 mm, acquisition matrix: 128, body receiver coil) and processed to generate segmented attenuation correction maps. Additional high-resolution breath-hold gradient echo sequences were obtained for anatomical co-registration of PET data. Since this study utilized whole-body PET/MRI, high-resolution dedicated brain MRI sequences were not acquired. Consequently, anatomical MRI images were not included in the analysis, and PET findings were evaluated independently. PET images were normalized to the template space and analyzed with a set of atlas regions.

### 2.3 Image analysis

The 18F-FDG brain metabolism was measured using PMOD software (version 4.201; PMOD Technologies LLC). A nuclear medicine physician with 7 years of experience (LB) trained a postdoctoral fellow (ZSV) to measure mean and maximum standardized uptake values (SUVmean and SUVmax, respectively) of the entire brain cortex, the prefrontal cortex, the cingulate gyrus, the hippocampus, caudate, thalamus, and angular gyrus. Brain regions were segmented using the automated PNEURO tool in PMOD software, which creates 3D volumes of interest, and measured SUV values within these regions. For bilateral subregions, SUV values were measured separately for the left and right hemispheres, and the mean of both sides was used for analysis to provide a comprehensive assessment of metabolic changes. As an exploratory analysis of lateralization, we additionally measured SUVmean and SUVmax separately in the right and left cortex, hippocampi, basal ganglia, and other bilateral brain regions. These subregions were selected based on prior literature demonstrating their susceptibility to HDMTX (Theruvath et al., 2017; Shrot et al., 2019; Baratto et al., 2024; Bhojwani et al., 2014).

### 2.4 Statistical analysis

SUV measurements for the whole cortex and six anatomical subregions were displayed as means and standard deviations. Statistical analyses were performed using a generalized linear regression model (GLM) to assess post-treatment changes in SUV values compared to baseline. For the whole cortex, differences in SUVmean and SUVmax between pre- and post-treatment scans were evaluated using GLM, with statistical significance set at *p* < 0.05. No Bonferroni correction was applied, as a single comparison was conducted for each metric.

Pre- and post-treatment SUV values across of six anatomical brain subregions (prefrontal cortex, cingulum, thalamus, caudate, angular gyrus, and hippocampus) were compared with a GLM, applying a Bonferroni correction for multiple comparisons. The adjusted significance threshold was calculated as *p* = 0.05/6 = 0.008. The GLM computed coefficient estimates with standard errors and 95% confidence intervals (CIs), derived from the parameter estimated covariance matrix. All statistical analyses were performed using Stata 18 (College Station, TX: StataCorp LLC).

## 3 Results

### 3.1 Whole brain cortex

#### 3.1.1 Short-term group

**Figure 3:**
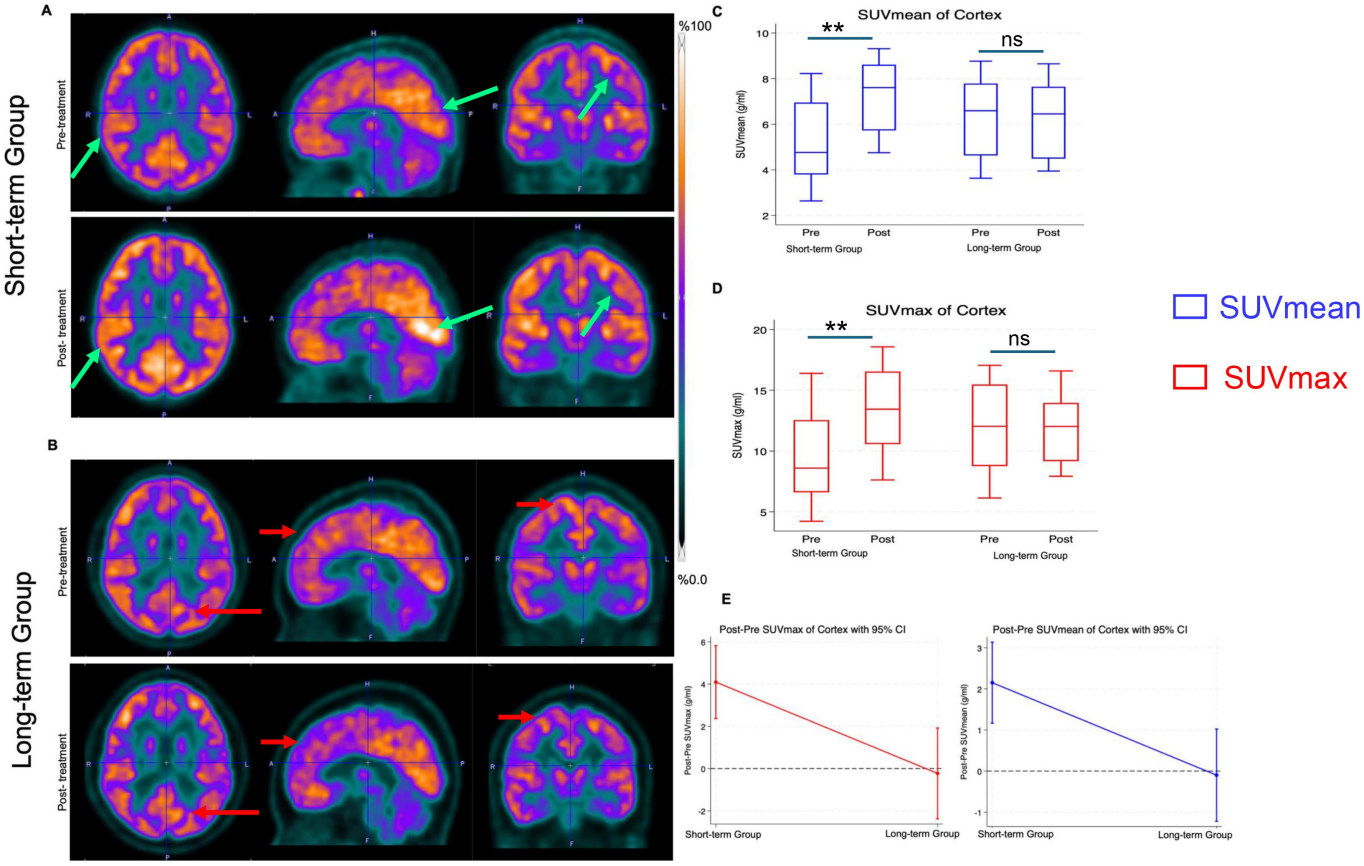
Brain ^18^F-FDG PET imaging before and after HDMTX. **(A)** Representative ^18^F-FDG PET scans of a pediatric cancer survivor before HDMTX therapy at 82 days after completion of HDMTX demonstrates increased FDG uptake of the whole cortex on the post-treatment scan (arrow) compared to the pre-treatment scan. **(B)** Representative ^18^F-FDG PET scans of a pediatric cancer survivor demonstrates no significant change in FDG uptake of the whole cortex (arrow) before HDMTX therapy and at 371 days after completion of HDMTX. **(C,D)** Mean SUV_mean_ (blue) and SUV_max_ (red) values and standard deviations of the whole cortex at baseline and at 1–3 months (short-term follow up, *n* = 11) or > 3 months (long term follow up, *n* = 8). (**E**) Differences between baseline and post-treatment SUV values of the whole cortex. Generalized linear regression model. Error bars represent the 95% confidence intervals. *p* < 0.001, ns = not significant.

Compared to baseline 18F-FDG PET scans before the start of HDMTX, patients in the short-term follow-up group exhibited a marked increase in FDG uptake across the whole cortex on post-treatment scans at 1–3 months after HDMTX (Figure 3A). Accordingly, whole cortex SUVmean after HDMTX (7.21 ± 1.51) was significantly higher compared to SUVmean at baseline (5.06 ± 1.62, *p* = 0.002) and whole cortex SUVmax after HDMTX (13.25 ± 3.35) was significantly higher compared to SUVmax at baseline (9.16 ± 3.33, *p* = 0.002) (Figures 3C,D). The brain metabolism of the whole cortex increased significantly by 2.15 for SUVmean (95% CI: 1.17–3.14, *p* < 0.001) and 4.09 for SUVmax (95% CI: 2.37–5.82, *p* < 0.001) (Figure 3E). In the short-term follow-up group (*n* = 11), chart review did not reveal any cases of overt methotrexate-induced neurotoxicity, such as seizures, stroke-like focal deficits, or encephalopathy. Most patients reported only mild, transient neurological complaints in the interval around the PET scan. Headache and fatigue were the most frequent symptoms, each occurring in 5/11 patients (45%). Blurred vision and dizziness were reported in 2/11 patients (18%) each, and isolated nausea, subjective memory difficulties, or transient spontaneous leg weakness were each documented in 1/11 patients (9%). Two patients (18%) had no documented neurological symptoms.

#### 3.1.2 Long-term group

Compared with baseline, the long-term group did not show a significant difference in whole-cortex FDG uptake (Figure 3B). Accordingly, SUVmean after HDMTX (6.30 ± 1.54) was not significantly different compared to SUVmean at baseline (6.31 ± 1.71, *p* = 0.1) and whole cortex SUVmax after HDMTX (11.58 ± 3.07) was not significantly different compared to SUVmax at baseline (12.01 ± 3.53, *p* = 0.834). Whole cortex SUVmean decreased by 0.10 (95% CI: −1.22–1.03, *p* = 0.864) and 0.23 for SUVmax (95% CI: −2.38–1.92, *p* = 0.834) (Figure 3E).

### 3.2 Subregional analysis

**Figure 4:**
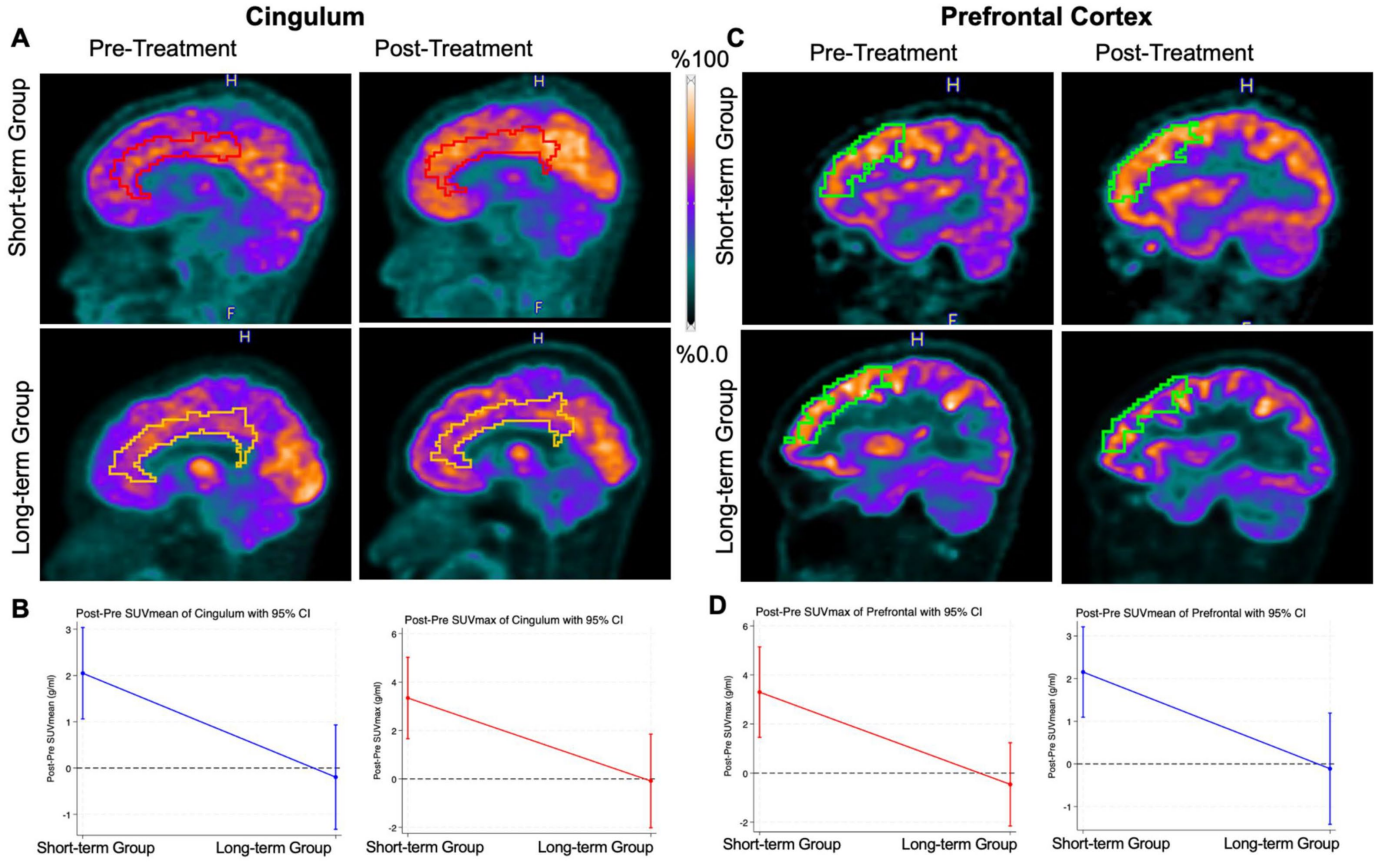
Representative PET images and quantification of FDG uptake in the cingulum and prefrontal cortex. **(A)** Representative ^18^F-FDG PET scans of a pediatric cancer survivor before HDMTX therapy at 39 days after completion of HDMTX (top row) demonstrates increased FDG uptake of the cingulum on the post-treatment scan compared to the pre-treatment scan. And, ^18^F-FDG PET scans of a pediatric cancer survivor demonstrates no significant change in FDG uptake of the cingulum before HDMTX therapy and at 371 days after completion of HDMTX (below row). **(B)** Differences between baseline and post-treatment SUV values of the cingulum at 1–3 months (short-term follow up, *n* = 11) and > 3 months (long term follow up, *n* = 8). Generalized linear regression model. Error bars represent the 95% confidence intervals. **(C)** Representative ^18^F-FDG PET scans of a pediatric cancer survivor before HDMTX therapy at 39 days after completion of HDMTX (top row) demonstrates increased FDG uptake of the prefrontal on the post-treatment scan compared to the pre-treatment scan. And,18F-FDG PET scans of a pediatric cancer survivor demonstrates no significant change in FDG uptake of the prefrontal before HDMTX therapy and at 246 days after completion of HDMTX (below row). **(D)** Differences between baseline and post-treatment SUV values of the prefrontal at 1–3 months (short-term follow up, *n* = 11) and > 3 months (long term follow up, *n* = 8). Generalized linear regression model. Error bars represent the 95% confidence intervals.

Further investigations of six anatomical subregions corroborated the results observed in the entire cortex: Compared to baseline 18F-FDG PET scans before start of HDMTX, patients in the short-term group demonstrated a significant increase in 18F-FDG uptake across all evaluated brain subregions, including the thalamus, caudate, angular gyrus, hippocampus, prefrontal cortex, and cingulum on 18F-FDG scans at 1–3 months after completion of HDMTX therapy (Table 2, Figures 4A–D). By comparison, the long-term group did not show significant change in FDG uptake of any examined subregion between baseline 18F-FDG scans and follow up scans at more than 3 months after end of HDMTX chemotherapy (Figures 4A–D). We did not observe any significant differences between left and right hemispheric SUVs at baseline or at short- or long-term follow-up. SUVmean and SUVmax of the whole cortex, hippocampi, basal ganglia, and other evaluated subregions were similar in the left and right hemispheres at all time points (all *p* > 0.05).

**Table 2:** Differences in SUV values at baseline and at 1–3 months (short-term follow up) and > 3 months (long-term follow up) after completion of HDMTX.

Subregion	Measurement	Group	Post-pre difference	95% CI	*p*-value
Caudate	SUVmean	Short-term	1.37	(0.57, 2.17)	0.001
		Long-term	−0.19	(−0.90, 0.53)	0.609
	SUVmax	Short-term	2.89	(1.40, 4.37)	<0.001
		Long-term	0.34	(−1.31, 1.99)	0.688
Angular	SUVmean	Short-term	2.33	(1.26, 3.39)	<0.001
		Long-term	0.02	(−1.25, 1.29)	0.976
	SUVmax	Short-term	3.27	(1.85, 4.69)	<0.001
		Long-term	0.12	(−1.90, 2.15)	0.905
Thalamus	SUVmean	Short-term	1.61	(0.85, 2.37)	<0.001
		Long-term	−0.19	(−1.09, 0.71)	0.682
	SUVmax	Short-term	2.7	(1.46, 3.94)	<0.001
		Long-term	0.04	(−1.66, 1.74)	0.963
Hippocampus	SUVmean	Short-term	0.95	(0.43, 1.47)	<0.001
		Long-term	−0.3	(−0.78, 0.18)	0.222
	SUVmax	Short-term	2.1	(0.99, 3.21)	<0.001
		Long-term	0.03	(−1.10, 1.16)	0.96
Prefrontal	SUVmean	Short-term	2.15	(1.10, 3.21)	<0.001
		Long-term	−0.11	(−1.42, 1.19)	0.867
	SUVmax	Short-term	3.3	(1.45, 5.14)	<0.001
		Long-term	−0.46	(−2.16, 1.24)	0.596
Cingulum	SUVmean	Short-term	2.05	(1.06, 3.04)	<0.001
		Long-term	−0.2	(−1.32, 0.93)	0.734
	SUVmax	Short-term	3.34	(1.66, 5.02)	<0.001
		Long-term	−0.08	(−2.02, 1.85)	0.932

Differences between baseline and post-treatment SUV values of six subregions at 1–3 months (short-term follow up, *n* = 11) and > 3 months (long term follow up, *n* = 8). Generalized linear regression model was applied and *p* value less than 0.008 was used as threshold for sub-region analysis with Bonferroni correction on multiple comparisons.

### 3.3 Case study

**Figure 5:**
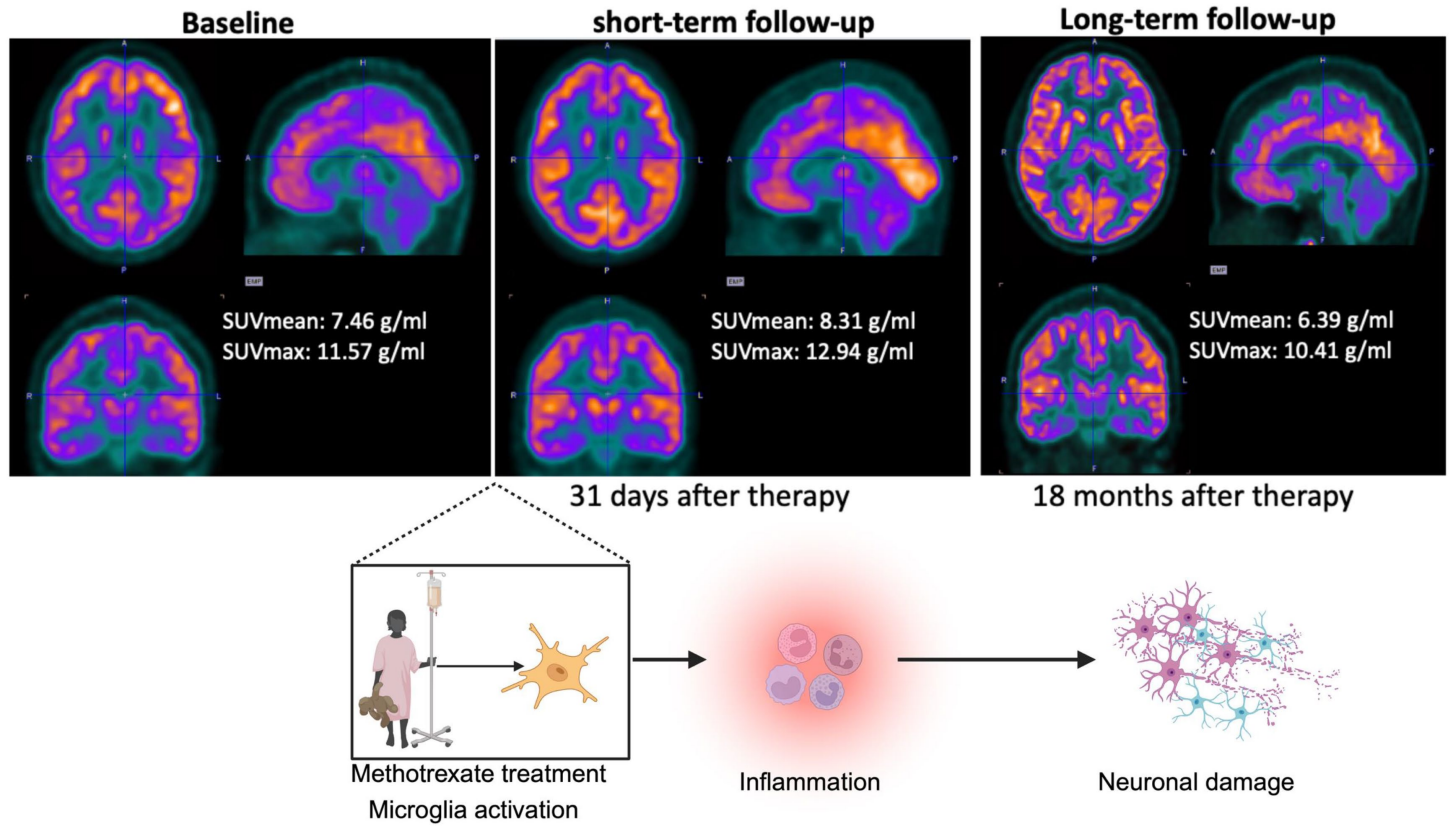
Representative PET images and mechanism alignment for a patient undergoing HDMTX therapy. The top panel shows FDG PET images at three distinct timelines: baseline (left), short-term follow-up at 31 days after completion of HDMTX (center), and long-term follow-up at 18 months after completion of HDMTX (right) in a 20-year old man. SUVmean and SUVmax are included at each stage, highlighting an increase in FDG uptake during the short-term follow-up, followed by a decline in the long-term follow-up. The bottom panel illustrates the proposed mechanism of HDMTX-induced changes in brain metabolism. Methotrexate treatment triggers microglial activation, many leading to acute inflammation, as observed during the short-term follow-up. Over time, chronic inflammation may contribute to neuronal damage and reduced metabolic activity, corresponding to the findings in the long-term follow-up images.

A 20-year-old male with diffuse large B-cell lymphoma (DLBCL), underwent both short-term (31 days) and long-term (18 months) 18F-FDG PET scans after end of HDMTX therapy. Compared to baseline, the brain cortex showed an increased 18F-FDG uptake on the short-term follow up scan and decreased 18F-FDG uptake on the long-term follow up scan (Figure 5). Specifically, whole brain cortex SUVmean was 7.46 g/mL at baseline, 8.31 g/mL at 1 month after HDMTX, and 6.39 g/mL at 18 months after HDMTX. SUVmax was 11.57 g/mL at baseline, 12.94 g/mL at short term follow up, and 10.41 g/mL at long term follow up. These findings suggest a possible pattern of an initial inflammatory response, as indicated by increased FDG uptake in the short-term follow-up, followed by a decline in metabolism on long-term imaging, which may be indicative of evolving neurotoxicity.

## 4 Discussion

Our findings demonstrated that brain metabolism on 18F-FDG PET at increased at 1–3 months after the end of HDMTX therapy, followed by normalization or decreased brain metabolism at more than 3 months after HDMTX therapy completion.

These results may reflect an initial inflammatory response of the brain, characterized by microglia activation, followed by normalization or neurotoxicity, described in basic science studies (Seigers et al., 2010; Gibson et al., 2019; Vazi et al., 2021).

The timing of MTX-related neuroinflammatory responses is likely variable and influenced by factors such as patient age, cumulative MTX exposure, folate metabolism, and co-existing medical conditions. Previous studies have documented long-term sequelae associated with HDMTX at >3 months post-treatment. Theruvath et al. (2017) found significant reductions in brain glucose metabolism of the cerebral cortex in pediatric cancer survivors on 18F-FDG PET at a median follow-up of 18.7 months after HDMTX therapy. Baratto et al. (2024) reported significant reductions in glucose metabolism of the prefrontal cortex and cingulum on 18F-FDG PET/MRI scans of pediatric cancer survivors at 1.2–10.6 years after HDMTX therapy. Zeller et al. (2013) reported significant volume loss of the cortical gray matter, white matter, and hippocampus on MRI scans of survivors of childhood acute lymphoblastic leukemia (ALL) at 10–42 years after HDMTX. Case-based imaging reports further support a multiphasic process: Eichler et al. (2007) described diffusion restriction appearing shortly after high-dose MTX, subsequently resolving, and being followed by new T2-weighted white-matter hyperintensity approximately 3.5 months later, consistent with delayed leukoencephalopathy. These data informed our choice of a 3-month threshold to distinguish short- from long-term follow-up but also underscore that individual trajectories may not align perfectly with this fixed time point.

Several investigators reported that abnormal imaging findings on MRI and PET scans after HDMTX correlated with impaired neurocognitive function. Berlin et al. (2020) reported that HDMTX-induced oligodendrocyte loss, quantified with diffusion tensor imaging (DTI), correlated with cognitive impairments in a rodent model. Baratto et al. (2024) reported that reduced metabolic activity of prefrontal cortex and cigulum correlated with significantly reduced executive function test scores in pediatric cancer survivors. Zeller et al. (2013) reported that volume loss of the cortical gray matter, white matter, and hippocampus on MRI scans, correlated with reduced processing speed, executive function, and verbal learning/memory in long-term survivors of childhood ALL.

Understanding this progression from neuroinflammation to neuronal damage and cognitive impairment is crucial, as the early inflammatory response may be amenable to anti-inflammatory therapies, potentially mitigating long-term neurocognitive effects. Although, given that FDG is a non-specific agent, it is difficult to attribute the increased uptake solely to neuroinflammation/microglial activation.

Sritawan et al. (2020) reported that anti-inflammatory treatment with Metformin, administered as a preventative treatment at 200 mg/kg/day and alongside MTX chemotherapy, alleviated memory and hippocampal neurogenesis decline induced by methotrexate chemotherapy in a rat model. Lyons et al. (2011) reported that Fluoxetine reversed memory impairment and reduction in proliferation and survival of hippocampal cells caused by methotrexate chemotherapy. Cordelli et al. (2017) reported that neurocognitive rehabilitation programs, which include cognitive exercises and psychosocial support, helped improve memory and attention deficits in pediatric patients receiving chemotherapy.

We recognize several limitations of our study. While our single-center design resulted in a relatively small patient cohort, it also ensured uniformity in imaging protocols for all participants. To confirm our findings, future research will require validation in a larger prospective cohort. Future research could strengthen the validation of medical imaging results by integrating blood or cerebrospinal fluid (CSF) biomarkers of inflammation, neurocognitive assessments, and electroencephalography (EEG) results. This would allow for correlations of imaging findings with functional outcomes. Patients suffering from chemotherapy-induced neurotoxicity demonstrated diffuse slowing of EEG brain activity (Santangelo et al., 2022). In addition, all patients were treated with multidrug chemotherapy regimens in which HDMTX was one component. Although we excluded patients who had received any form of cranial or craniospinal irradiation to minimize confounding effects of brain radiotherapy, we cannot completely rule out contributions from other systemic agents to the observed changes in brain metabolism. Nevertheless, HDMTX remains the agent most strongly associated with neurotoxicity in this clinical setting and was the primary exposure of interest in our analysis. In the current study, none of the patients in our cohort developed overt methotrexate-induced neurotoxicity, such as seizures, stroke-like focal deficits, or encephalopathy around the time of PET imaging; only mild, transient symptoms such as headache, fatigue, dizziness, blurred vision. Consequently, our study cannot determine whether patients with clinically significant neurotoxicity exhibit different patterns or magnitudes of FDG SUV change compared with asymptomatic or mildly symptomatic patients. Future multi-center studies that specifically include patients with methotrexate-related stroke-like episodes, seizures, or encephalopathy, together with standardized neurological, EEG, and neurocognitive assessments, will be needed to clarify whether FDG PET can help distinguish clinically overt neurotoxicity from subclinical metabolic alterations. Long-term follow-up scans revealed decreased brain metabolism in some patients; however, the small sample size precluded determining whether these decreases correlated with neurocognitive impairment. Furthermore, experimental studies could investigate whether anti-inflammatory treatment resolves the short-term inflammatory response seen on FDG PET and improves long-term outcomes, potentially making FDG PET a useful tool for monitoring the efficacy of such treatment.

## 5 Conclusion

HDMTX therapy induces a biphasic metabolic response in the brain, with an increase in FDG metabolism of the cortex at 1–3 months after completion of HDMTX therapy, likely reflecting inflammatory microglial activation, followed by a subsequent non-significant decline in metabolism during long-term follow-up. 18F-FDG PET/MRI could be used to identify potential candidates for anti-inflammatory therapies and evaluating the efficacy of these therapies.

## Data Availability

The original contributions presented in the study are included in the article/supplementary material, further inquiries can be directed to the corresponding author.
